# When Sarcoid Squeezes and Weakens: A Dual-Mechanism Cause of Severe Esophageal Dilation

**DOI:** 10.14309/crj.0000000000002158

**Published:** 2026-06-02

**Authors:** Rayhan Karimi, Zachary Makovich, Lee Sigmon

**Affiliations:** 1 Department of Gastroenterology, Atrium Health, Charlotte, NC

## CASE REPORT

**Figure 1.**
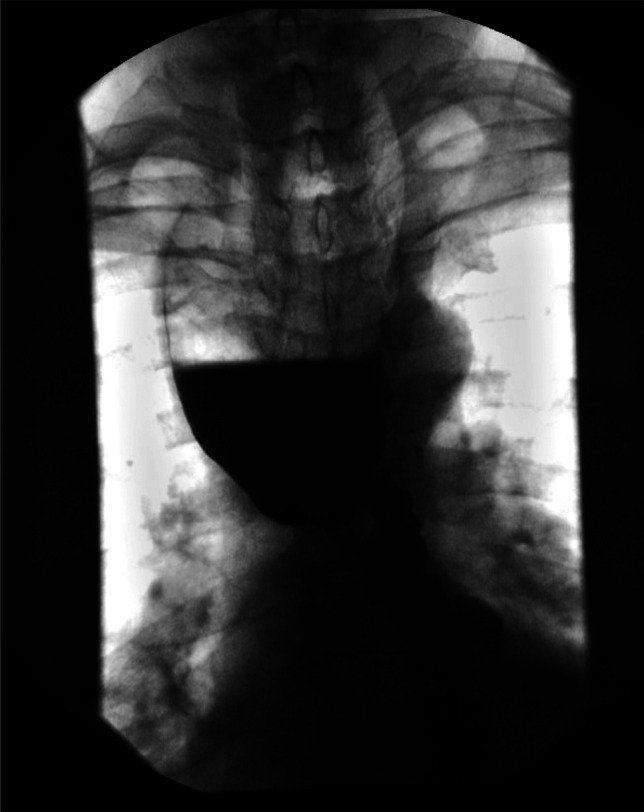
Barium esophagram showing the markedly distended upper thoracic esophagus with abrupt transitional narrowing at the level of the carina.

**Figure 2.**
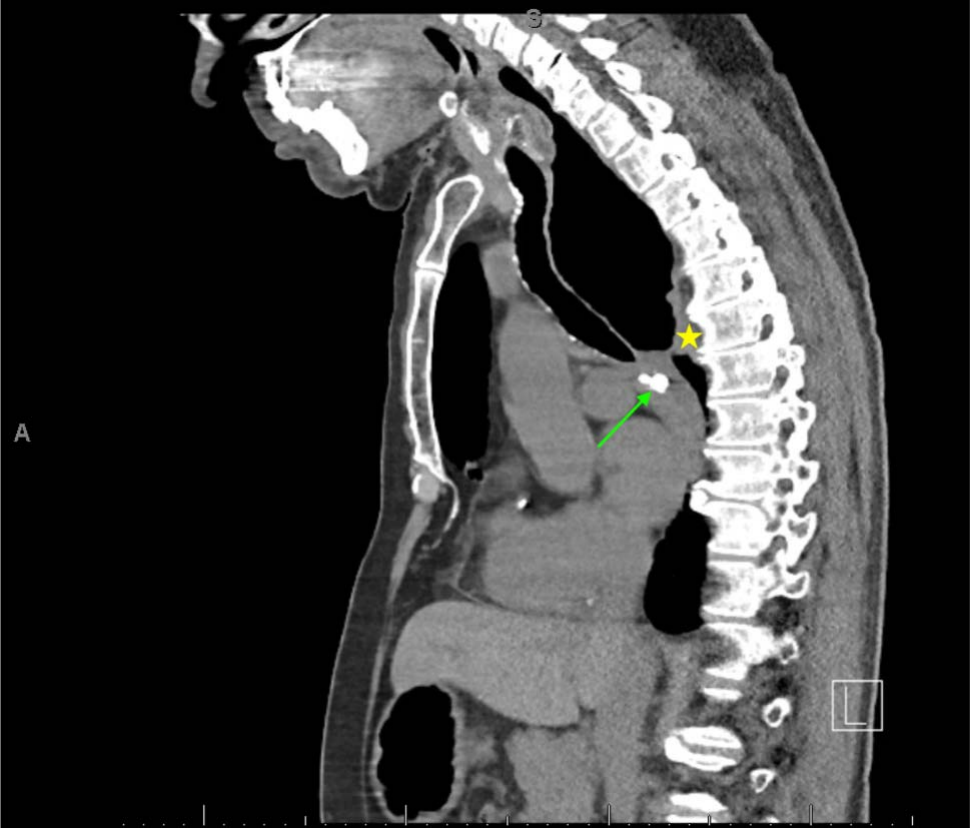
Diffuse dilation of the thoracic esophagus with abrupt narrowing and wall thickening at the level of the gastroesophageal junction (yellow star). Extrinsic compression and prominent upstream dilation where there is calcified mediastinal adenopathy (green arrow).

**Figure 3.**
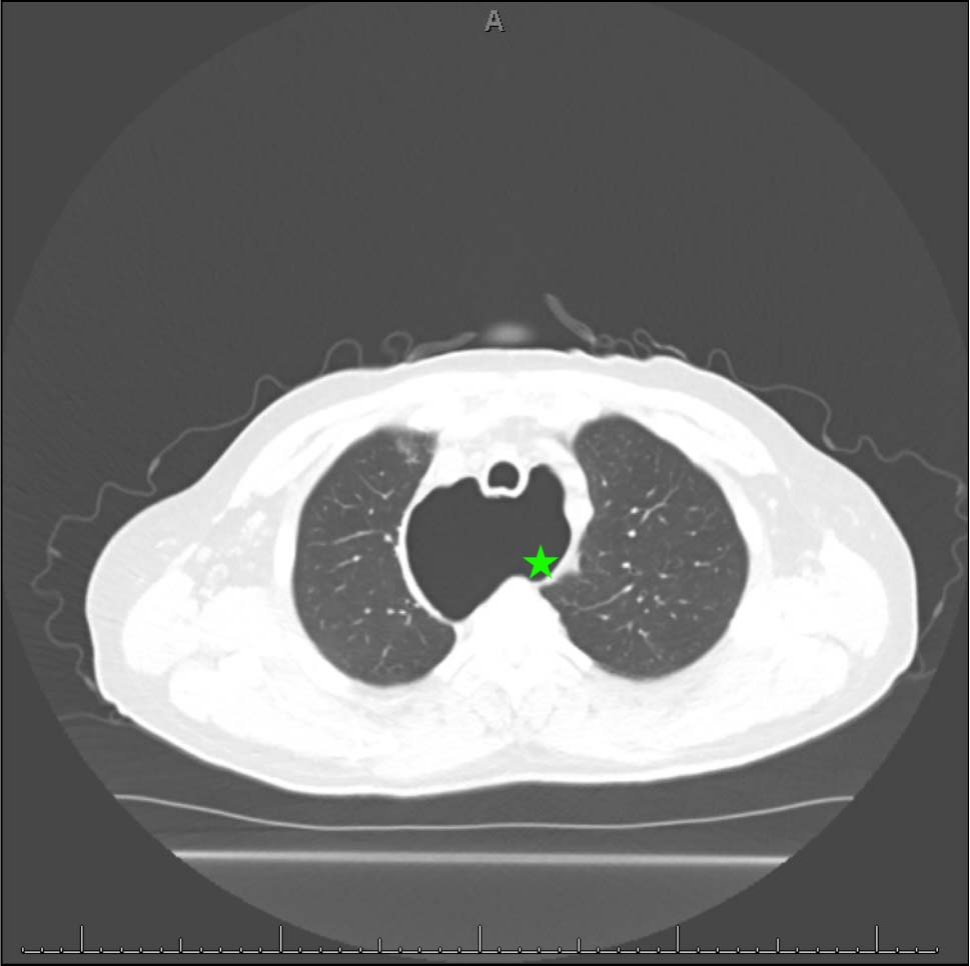
Axial computed tomography esophagram showing marked thoracic esophageal dilation (green star).

**Figure 4.**
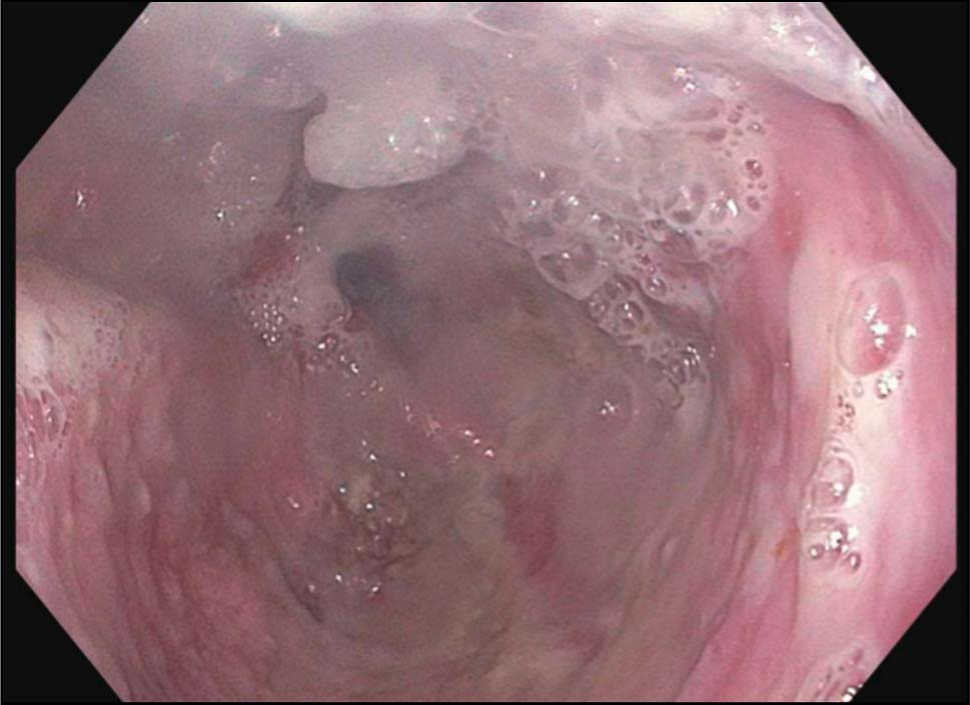
Esophagogastroduodenoscopy showing the fluid-filled esophagus with severe dilation and maceration from stasis of retained products.

Sarcoidosis is a multisystem granulomatous disease most commonly affecting lungs and mediastinal lymph nodes. Gastrointestinal involvement is rare, and esophageal disease is particularly uncommon, typically presenting as achalasia-like dysmotility due to myenteric plexus involvement.^1^ We report an 82-year-old man with recurrent sarcoidosis presenting with progressive dysphagia and marked esophageal dilation. Imaging demonstrated severe dilation with subcarinal calcified lymphadenopathy causing extrinsic compression near the gastroesophageal junction (Figures 1–3). Endoscopy showed a dilated fluid-filled esophagus without intrinsic obstruction (Figure 4). Biopsy confirmed non-necrotizing granulomas. High-resolution manometry demonstrated preserved lower esophageal sphincter (LES) relaxation with ineffective esophageal motility in >50% of swallows.

Similar dual-mechanism presentations are rarely described in the literature, with most reports attributing dilation to either achalasia-like motor failure or isolated extrinsic compression.^2^ In this case, ineffective esophageal motility contributed significantly to dilation despite normal LES relaxation by preventing effective peristaltic clearance of swallowed material. This led to persistent intraluminal stasis, increased luminal distension, and progressive esophageal dilation, outflow at the LES was not obstructed. Concurrent extrinsic compression further impaired bolus transit by creating a fixed narrowing, amplifying upstream stasis.^3^ The combination of impaired propulsion and partial mechanical resistance resulted in functional esophageal obstruction without classic achalasia physiology. Recognition of mechanisms is important for diagnosis sarcoidosis-related dysphagia clinical.

## DISCLOSURES

Author contributions: All authors had substantial contributions to conception of work, drafting the work, and accountability for all aspects of the work. R. Karimi is the article guarantor.

Financial disclosure: None to report.

Informed consent was obtained for this case report.
